# Microtubule-associated protein 1b is required for shaping the neural tube

**DOI:** 10.1186/s13064-015-0056-4

**Published:** 2016-01-18

**Authors:** Pradeepa Jayachandran, Valerie N. Olmo, Stephanie P. Sanchez, Rebecca J. McFarland, Eudorah Vital, Jonathan M. Werner, Elim Hong, Neus Sanchez-Alberola, Aleksey Molodstov, Rachel M. Brewster

**Affiliations:** Department of Biological Sciences, University of Maryland Baltimore County, Baltimore, MD USA; Institut de Biologie Paris Seine-Laboratoire Neuroscience Paris Seine INSERM UMRS 1130, CNRS UMR 8246, UPMC UM 118 Université Pierre et Marie Curie, Paris, France

## Abstract

**Background:**

Shaping of the neural tube, the precursor of the brain and spinal cord, involves narrowing and elongation of the neural tissue, concomitantly with other morphogenetic changes that contribue to this process. In zebrafish, medial displacement of neural cells (neural convergence or NC), which drives the infolding and narrowing of the neural ectoderm, is mediated by polarized migration and cell elongation towards the dorsal midline. Failure to undergo proper NC results in severe neural tube defects, yet the molecular underpinnings of this process remain poorly understood.

**Results:**

We investigated here the role of the microtubule (MT) cytoskeleton in mediating NC in zebrafish embryos using the MT destabilizing and hyperstabilizing drugs nocodazole and paclitaxel respectively. We found that MTs undergo major changes in organization and stability during neurulation and are required for the timely completion of NC by promoting cell elongation and polarity. We next examined the role of Microtubule-associated protein 1B (Map1b), previously shown to promote MT dynamicity in axons. *map1b* is expressed earlier than previously reported, in the developing neural tube and underlying mesoderm. Loss of Map1b function using morpholinos (MOs) or δMap1b (encoding a truncated Map1b protein product) resulted in delayed NC and duplication of the neural tube, a defect associated with impaired NC. We observed a loss of stable MTs in these embryos that is likely to contribute to the NC defect. Lastly, we found that Map1b mediates cell elongation in a cell autonomous manner and polarized protrusive activity, two cell behaviors that underlie NC and are MT-dependent.

**Conclusions:**

Together, these data highlight the importance of MTs in the early morphogenetic movements that shape the neural tube and reveal a novel role for the MT regulator Map1b in mediating cell elongation and polarized cell movement in neural progenitor cells.

**Electronic supplementary material:**

The online version of this article (doi:10.1186/s13064-015-0056-4) contains supplementary material, which is available to authorized users.

## Background

The neural tube, the precursor of the central nervous system, derives from the neurectoderm through a process known as neurulation. In anterior regions of mouse, chick and *Xenopus* embryos, conserved aspects of this process entail thickening of the neural ectoderm to shape the neural plate, elevation of the edges of the neural plate to form neural folds and convergent extension of the neural plate that narrows and elongates the neural ectoderm [1–4] and contributes to neural groove formation. The neural folds on either side of the neural plate eventually fuse at the dorsal midline and separate from the overlying non-neural ectoderm to shape the neural tube [5]. Mechanisms of teleost neurulation are often thought to diverge from primary neurulation due to the initial formation of a solid rod (and hence absence of a neural groove), which only later cavitates to give rise to a neural tube [6]. A common misconception is that the neural rod is assembled from the coalescence of neurectodermal cells that exhibit mesenchymal properties (reviewed in [3]), akin to secondary neurulation in mammals. However, closer examination of this process in zebrafish revealed that the neural tube derives in fact from a bilayered neural plate, albeit incompletely epithelialized, that infolds as a continuous sheet. The two sides of the neural plate are closely juxtaposed during infolding, explaining the absence of a neural groove. Thus, medio-lateral positions of cells in the deep layer of the neural plate correlate with dorso-ventral positions in the neural tube [7, 8]. In this regard, neural tube formation in zebrafish is similar to primary neurulation in mammals, which also entails the folding of an epithelialized neural plate.

As in other vertebrates [9-11], the zebrafish neural plate undergoes neural convergence and extension. However, in zebrafish, narrowing and elongation of the neural anlage is not limited to the neural plate stage, since convergence also drives infolding of the neural plate to shape the neural rod and extension occurs concomitantly with this event. This later convergence event (referred to henceforth as NC, for neural convergence) is driven by polarized migration towards the dorsal midline and cell elongation along the medio-lateral (prospective apico-basal) axis. Failure to undergo proper NC, as a consequence of disruption of the planar cell polarity (PCP) pathway, results in severe neural tube defects in zebrafish [12], highlighting the importance of this early stage of neural tube formation.

The cellular mechanisms underlying NC were first revealed in *Xenopus* and zebrafish, owing to early access and transparency (zebrafish) of the embryo. In *Xenopus*, explant assays have revealed that migration of deep neural cells in the medial neural plate is mediated by monopolar protrusions (filopodia and lamellipodia) directed towards the midline [11, 13, 14]. We have previously demonstrated that cells in the zebrafish neural plate also extend medially-oriented protrusions and elongate as they converge towards the midline [8]. Narrowing of the neural plate in mice involves cell elongation [15] and cellular rearrangements [10, 16] that are driven by polarized apical boundary rearrangement and bipolar protrusive activity at the basal pole of cells [9]. Thus, the ability of neuroepithelial cells to form polarized protrusions appears to be an essential and conserved aspect of neural tube morphogenesis, the molecular underpinnings of which remain poorly understood.

Many inroads have been made in understanding how the microtubule (MT) network contributes to cell polarity during migration [17]. MTs are dynamic heteropolymers of α- and β-tubulin, existing in alternating states of active polymerization and depolymerization known as dynamic instability [18, 19]. These cytoskeletal elements establish the position of cortical polarity (manifested as actin-rich lamellipodia in migrating cells) via multiple pathways [20, 21]. Key to MT-mediated establishment of cellular asymmetry is the polarized (radial) organization of these structures, with slow-growing minus-ends anchored at the centrosome and the faster growing plus-ends clustered at the leading edge, adjacent to the cell cortex [22]. In addition to their role in cell migration, dynamic MTs play an active role in cell elongation and maintenance of homeostatic length [23]. The role of stable MTs in cellular dynamics is less well established.

MT stability and dynamics are regulated in part by microtubule-associated proteins (MAPs). Members of the MAP1 family bind along the entire MT lattice. MAP1B, a founding member of this family, is post-translationally cleaved into a heavy chain (HC) and a light chain (LC1) [24]. The heavy chain contains domains for actin, MTs and LC1 binding [25–27] and can therefore crosslink MTs and microfilaments [28, 29]. The light chain also binds MTs and actin and regulates the cytoskeleton [30, 31]. MAP1B proteins were first identified based on their MT-stabilizing properties [31–33]. However, unlike tau, MAP1B preferably associates with dynamic (tyrosinated) MTs, helping to maintain a pool of dynamic MTs required for axonal elongation [34, 35]. This activity of MAP1B is controlled by several kinases, including Glycogen synthase kinase-3β (GSK-3β), which increases MAP1B MT binding and dynamicity [36]. The poor MT stabilizing properties of MAP1B combined with its ability to promote MT dynamics, suggest MAP1B function differs from the other MAPs [35]. *MAP1B* is also expressed prior to other members of this family in the nervous system [37–40], as it is observed in neuronal progenitors prior to their last mitotic division [41]. Despite this early expression and function in promoting MT dynamics, MAP1B has not been implicated in early stages of neural tube development.

We investigate here whether zebrafish Map1b plays a role in the polarized cell movements that shape the neural rod during NC. Our studies reveal that MTs undergo major changes during neural tube formation, as they become progressively more stable and elongated. The perturbation of cell elongation and polarized migration following nocodazole and paclitaxel treatments suggests that the regulation of MT stability during NC is essential for proper completion of this process. To gain insight into underlying mechanism, we characterized the function of Map1b, previously shown to promote MT dynamicity in axons. *map1b* is expressed earlier than previously reported, in the developing neural tube and underlying mesoderm. Loss of Map1b function using morpholinos (MOs) or δMap1b, encoding a truncated Map1b protein product, resulted in delayed NC and duplication of the neural tube, a defect previously observed in PCP mutants in which NC is also defective [12]. We observed a loss of stable MTs in these embryos that is likely to contribute to the NC defect. Lastly, we reveal that Map1b mediates cell elongation in a cell autonomous manner and polarized protrusive activity, two cell behaviors that underlie NC and are MT-dependent. Together, these data highlight the importance of MTs in the early morphogenetic movements that shape the neural tube and reveal a novel role for the MT regulator Map1b in mediating cell elongation and polarized cell movement in neural progenitor cells.

## Results

### Microtubules undergo dramatic changes during neurulation

**Fig. 1:**
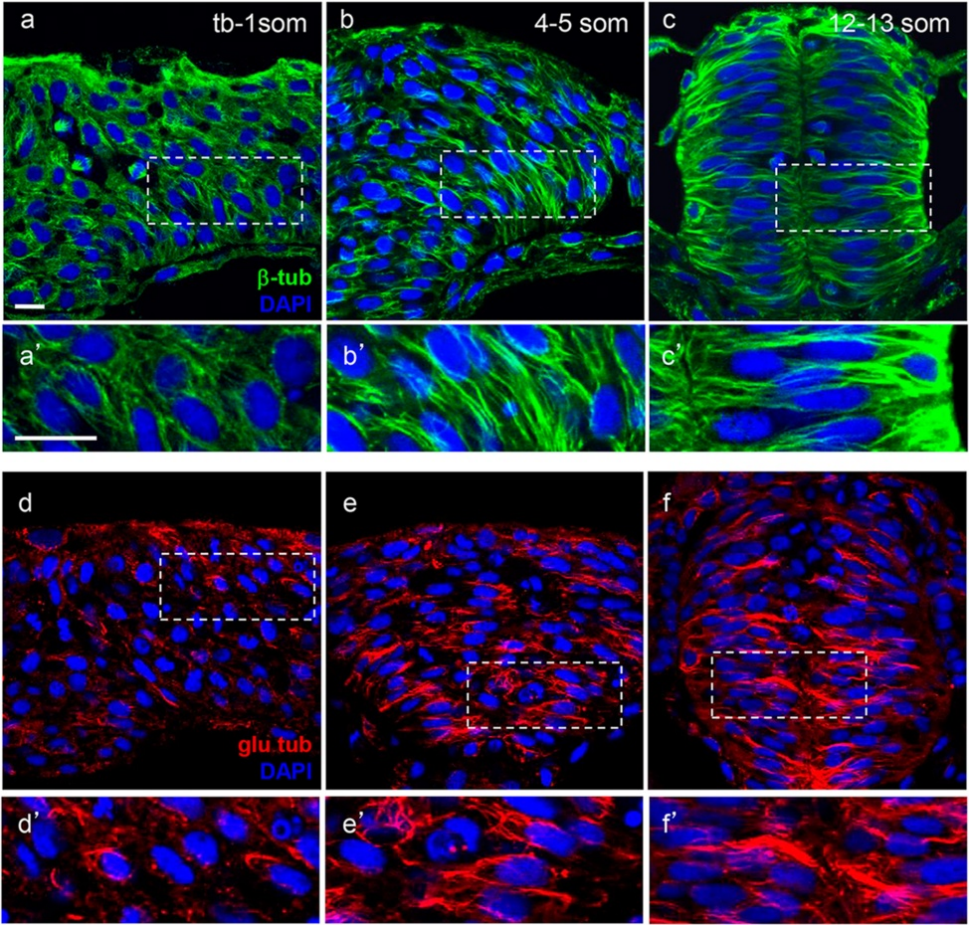
Microtubules become increasingly stabilized during neurulation. Hindbrain sections of embryos at the neural plate (tb-1 som) (**a**, *a’*, **d**, *d’*), neural keel (4–5 som) (**b**, *b’*, **e**, *e’*) and neural rod (12–13 som) (**c**, *c’*, **f**, *f’*) stages immunolabeled with anti-β-tub (total MTs) in green (**a**–*c’*) and anti-glu-tub (detyrosinated MTs) in red (**d**–*f’*). (*a’*–*c’*) and (*d’*–*f’*) Higher magnification of boxed areas in (**a**–**c**) and (**d**–**f**), respectively. Scale bars: 10 μm

During early stages of neurulation, MTs appear to undergo global morphological changes. Immunolabeling with anti-β−tubulin (anti-β-tub, a marker for the total MT population) revealed that at the neural plate stage (tb-1 som), when neural cells extend polarized protrusions towards the midline, MTs are distributed throughout the cytosol (Fig. 1a, a’), consistent with the radial organization we previously reported [42]. In contrast, at the neural keel stage (so named because of the keel shape adopted by the neural tissue as it transition from a neural plate to a neural rod, 4–5 som) and neural rod (12–13 som) stage, MTs organize into long linear arrays (bundles), which align along the future apico-basal axis of neural cells, coincident with epithelialization that occurs following NC (Fig. 1b–c’) [43].

The organizational changes observed in MTs suggest that they become increasingly stable as neurulation progresses. In order to investigate the distribution of stable MTs at different stages of neurulation, embryos were immunolabeled with anti-detyrosinated-tubulin (glu-tub). Glu-tub antibodies recognize stable, detyrosinated MTs by binding to the exposed carboxy-terminal glutamic acid of α-tubulin (α-tub) in MT polymers [44, 45]. At the neural plate stage, glu-tub labeling is diffuse and more punctate than β-tub (Fig. 1d, d’). However, by the neural keel stage (Fig. 1e, e’) detyrosinated MTs organize into linear structures, which become more accentuated at the neural rod stage (Fig. 1f, f’). The distribution of glu-tub labeling thus implies that detyrosination of MTs occurs initially in discrete foci along the MT polymers that subsequently expand to include the entire polymer. These observations suggest that stable MTs increase over time, reaching elevated levels in the epithelialized neural tube. To quantify the relative abundance of stable MTs, we analyzed the ratio of glu-tub (stable MTs) to α-tub (total MTs) at the neural plate (tb), neural keel and neural rod stages and found this ratio to be highest at the neural rod stage (Additional File 1: Figure S1).

**Fig. 2:**
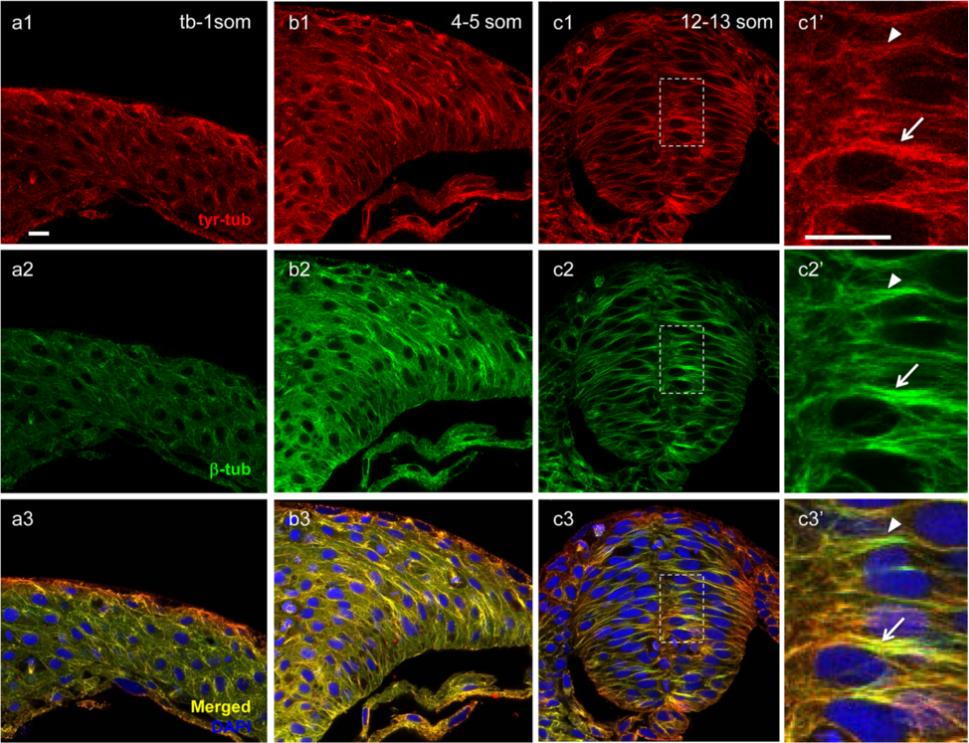
Distribution of dynamic microtubules during neurulation. Hindbrain sections of embryos at the neural plate (tb-1 som) (**a1**–**a3**), neural keel (4–5 som) (**b1**–**b3**) and neural rod (12–13 som) (**c1**–*c3’*) stages immunolabeled with anti-tyr-tub (dynamic MTs) in red (**a1**, **b1**, **c1**, *c1’*), anti-β-tub (total MTs) in green (**a2**, **b2**, **c2**, *c2’*). (**a3**, **b3**, **c3**, *c3’*) Red-Green overlay (yellow) of images in (**a1**-*c2*’) with nuclei labeled in blue using DAPI. (*c1’*-*c3’*) Higher magnification of boxed areas in (**c1**–**c3**). Arrows indicate high overlap between anti-tyr-tub and anti-β-tub; arrowheads indicate area of reduced overlap between these two markers. Scale bars: 10 μm

We next analyzed the distribution of dynamic MTs using an antibody that specifically recognizes the tyrosinated form of α-tubulin (anti-tyr-tub) [46]. In contrast to the spotty distribution of glu-tub at the neural plate and neural keel stages, tyr-tub was abundant and appeared to near fully overlap with the bulk of MTs labeled with α β-tub (Fig. 2a1–a3, b1–b3), with the exception of discrete puncta of α tyr-tub labeling that may correspond to depolimerized tyrosinated tubulin (Fig. 2b3). At the neural rod stage, the overlap remained extensive but some segments of MT bundles were more intensely labeled with anti- β-tub than with anti- tyr-tub (Fig. 2c3, c3’). These regions may coincide with areas of MT stabilization (arrowhead in Fig. 2c3’). Thus, dynamic MTs represent the bulk of the MT population during NC, while stable MTs steadily increase over time.

### Microtubules are required for NC

The striking increase in the levels of detyrosinated MTs suggests that the stability of these cytoskeletal elements is regulated during neural tube development and likely to be important for neural tube morphogenesis. To test this, we treated early neural stage (2–3 som) embryos with nocodazole (17 and 32 μM) or paclitaxel (50 μM), which destabilize and hyperstabilize MTs respectively, and analyzed the effect of these drugs on cell behaviors at the neural keel stage (4–5 som). The efficacy of these drugs was first confirmed by immunolabeling with anti-β-tub, which revealed that the linear organization of MTs was disrupted following both treatments (Additional file 2: Figure S2).

**Fig. 3:**
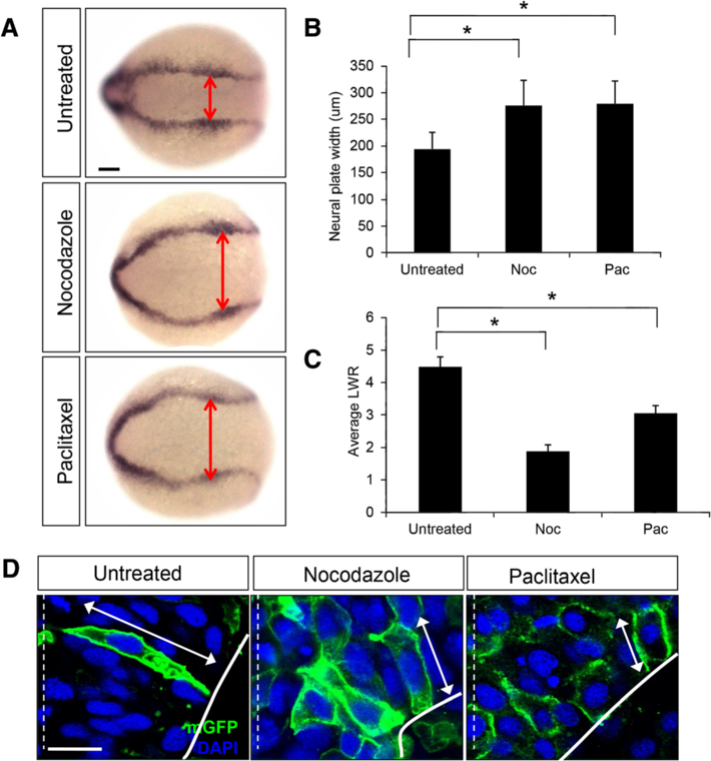
Regulation of microtubule dynamics is required for NC. **a** Dorsal views of untreated, nocodazole-treated (5 μg/ml) and paclitaxel-treated (50 μM) embryos labeled by *in situ* hybridization with the *dlx3* riboprobe. Double red arrowheads indicate the width of the neural plate. Scale bar: 100 μm . **b** Quantification of the neural plate width (μm) in control (untreated) and drug-treated embryos. (*) indicates statistical significance (*P* <0.001 for untreated vs nocodazole and untreated vs paclitaxel) using a Kruskal-Wallis test followed by Dunn’s post-hoc test. **c** Quantification of the length-to-width (LWR) ratio of mGFP-labeled cells in control (untreated), nocodazole-treated, and paclitaxel-treated embryos at the 4–5 som stage. (*) indicates statistical significance (*P* <0.001 for untreated vs nocodazole and *P* <0.01 for untreated vs paclitaxel) using a Kruskal-Wallis test followed by Dunn’s post-hoc test. **d** Hindbrain sections of 4–5 som control (untreated), nocodazole-treated and paclitaxel-treated embryos mosaically expressing mGFP (green). Nuclei are labeled in blue with DAPI. Double arrows indicate cell length. The dotted white line represents the midline. Scale bar: 10 μm

To analyze the effect of altering MT stability on NC following nocodazole or paclitaxel treatments, the width of the neural plate was assessed using *dlx3*, a gene expressed at the border of the neural and non neural ectoderm. While untreated embryos displayed no NC defects, the neural plates of nocodazole and paclitaxel-treated embryos were abnormally wide (A,B) (untreated embryos: 194 μm ± 31 μm, *n*= 38 embryos; nocodazole-treated (17μΜ): 276 μm ± 47 μm, *n* = 34 embryos; paclitaxel-treated (50 μM): 279 μm ± 47 μm, *n* = 42 embryos). In order to investigate the underlying cellular cause for the NC defects, embryos mosaically expressing cell-surface Green Fluorescent Protein (mGFP) were exposed to nocodazole (17 and 32 μM) or paclitaxel (50 μM) and imaged in the hindbrain region at the neural keel (4–5 som) stage. Cells were significantly shortened following treatment with nocodazole and paclitaxel (Fig. 3c), as determined by length-to-width (LWR) ratio measurements (LWR of untreated cells: 4.48 ± 0.3, *n* = 26 cells from 7 embryos; LWR of nocodazole-treated cells 1.9 ± 0.19, *n* = 18 cells from 4 embryos; LWR of paclitaxel-treated cells: 3.0 ± 0.24, *n* = 30 cells from 7 embryos) (Fig. 3D), indicating sensitivity to perturbations of the MT network that either destabilize or hyperstabilize MTs.

In order to investigate whether MTs play a role in polarized cell migration, time-lapse confocal imaging was carried out at the neural plate (tb-1 som) stage using control and nocodazole-treated embryos mosaically expressing mGFP. Cells from untreated embryos exhibit an elongated appearance with protrusions oriented medially, as previously described [8]. In contrast, cells from nocodazole-treated embryos were rounded in shape and failed to migrate in a directional manner, as a result of randomized membrane protrusions (Additional files 3 and 4). This observation suggests that MTs are not required for the formation of protrusions but are rather implicated in the proper polarization of these extensions.

Together these findings identify MTs as key mediators of cell elongation and polarized cell movement during NC. They further suggest that regulation of MT stability is tightly controlled during early development, pointing to a potential role for microtubule-associated proteins (MAPs) in this process.

### Zebrafish *map1b* is expressed in the developing neural tube

Mammalian MAP1B is one of the earliest MAPs to be expressed in the developing nervous system and hence a good candidate for mediating early morphogenetic movements during neural tube formation. Gene ontology analysis revealed a high level of sequence similarity between zebrafish Map1b and its orthologues in chick, mouse, rat and human. The regions of highest conservation (98 % identity) comprise a stretch of 550 amino acids in the N-terminus and 120 amino acids in the C-terminus [31]. Mammalian MAP1B contains two MT-binding domains, each composed of multiple repeats of KKEE or KKEI/V motifs [26, 30]. Domain analysis of zebrafish Map1b revealed the presence of the conserved KKE signature repeats at the N-terminus, in the region encoding the heavy chain. Furthermore, synteny analysis showed that zebrafish *map1b* is located in a conserved region of the genome.

**Fig. 4:**
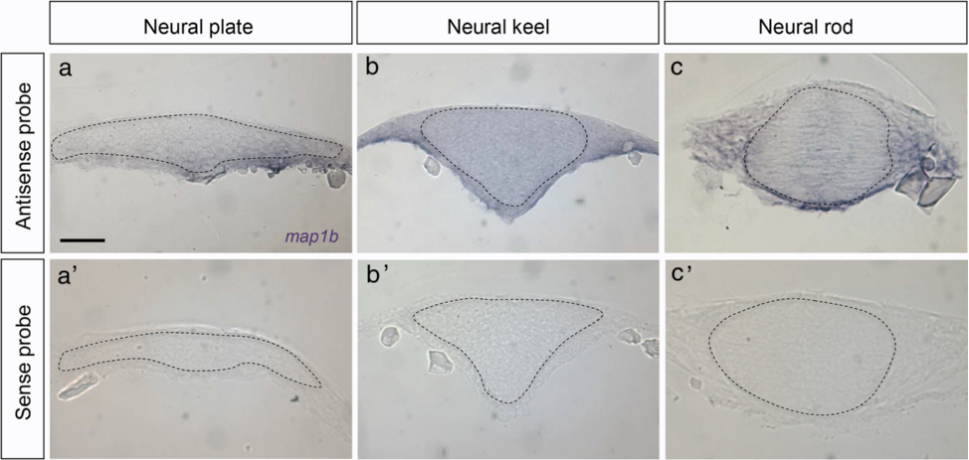
*map1b* mRNA distribution. Expression of *map1b* mRNA in hindbrain sections detected by *in situ* hybridization using anti-sense (**a**, **b** and **c**) or sense (**a’**, **b’** and **c’**) probes. (**a**, **a’**) neural plate, (**b**, **b’**) neural keel, (**c**, **c’**) neural rod stage embryos. The neural tissue is delineated by a dotted line. Scale bar: 20 μm

In order to determine whether zebrafish *map1b* is expressed during neurulation, we performed wholemount *in situ* hybridization. We observed that *map1b* is broadly distributed at the neural plate (tb-1som), neural keel (4–5 som) and neural rod (8–10 som) stages (Fig. 4a–c). Its expression appears to be in a gradient that is highest in the mesoderm at the neural plate stage (Fig. 4a). By the neural keel and rod stages the level of *map1b* increases and expression expands dorsally, as *map1b* is present throughout the developing neural tube (Fig. 4b, c). To confirm that the signal observed at these developmental stages is specific, *in situ* hybridization using a sense riboprobe was also performed. No labeling was observed with the latter (Fig. 4a’–c’).

These observations indicate that *map1b* is expressed earlier than previously reported, in undifferentiated neural progenitor cells undergoing NC.

### Depletion of Map1b causes NC defects

**Fig. 5:**
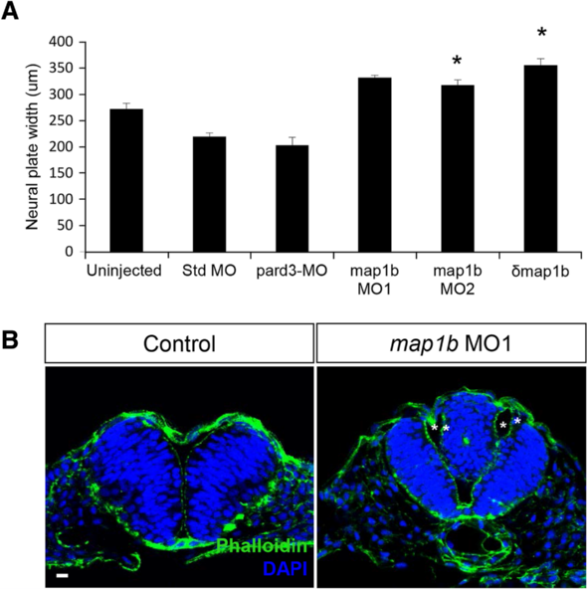
*map1b* depletion causes NC defects. **a** Quantification of the width of the neural plate (μm). (*) Indicates statistical significance (*P* <0.01 for uninjected vs *map1b* MO2; *P* <0.001 for uninjected vs *δmap1b*-injected) using a Kruskal-Wallis test followed by Dunn’s post-hoc test. **b** Hindbrain sections of uninjected and *map1b* MO1-injected embryos at 24hpf, labeled with Phalloidin (cortical actin, green) and DAPI (nuclei, blue). Asterisks indicate the ventricles of the duplicated neural tube. Scale bar: 10 μm

We next tested whether *map1b* is required for NC by performing functional studies using two splice-blocking MOs (*map1b* MO1 and *map1b* MO2, Additional file 5: Figure S3). RT-PCR analysis confirmed that these MOs block *map1b* mRNA splicing (Additional file 5: Figure S3). We observed that the neural plate of *map1b* MO1 (10 ng)- and *map1b* MO2 (4 and 10 ng)-injected embryos were significantly wider than those of uninjected and standard control MO (4 ng)-injected embryos (uninjected: 272 μm ± 11 μm, *n* = 58 embryos; standard MO: 220 μm ± 7 μm, *n* = 29 embryos; *map1b* MO1: 332 μm ± 15 μm, *n* = 8 embryos; *map1b* MO2: 319 μm ± 10 μm, *n* = 68 embryo; Fig. 5a), suggesting that *map1b* is required for NC. A MO targeting *pard3* (10 ng), a gene implicated in later aspects of neural tube development [42, 47], was used as an additional negative control and confirmed to not cause an NC defect (*pard3*-MO: 200 ± 15 μm, *n*= 14 embryos; Fig. 5a). To further confirm these results, a translation-blocking *map1b* MO (MO3) was designed, but was found to be less effective than the splice-blocking MO1 at producing a widened neural plate phenotype. However, co-injection of MO3 (10 ng) with lower concentrations of *map1b* MO1 (4 ng) resulted in a wider neural plate, which was not observed in embryos injected with *map1b* MO1 alone at the suboptimal concentration (uninjected embryos: 165 ± 21 μm, *n* = 10 embryos; *map1b* MO1 (4 ng): 145 ± 24 μm, *n* = 13 embryos; *map1b* MO3 (10 ng): 212 ± 23 μm, *n* = 26 embryos; *map1b* MO1 (4 ng) + MO3 (10 ng); 291 ± 29 μm, *n* = 24 embryos) (Additional file 5: Figure S3). Therefore, we conclude that the neural plate widening we observe is most likely due to loss of *map1b* rather than an off-target effect of the MOs.

The MT-binding affinity and activity of Map1b is known to be modulated by several kinases, including Gsk3β [48–50]. Consistent with this model, we observed a widened neural plate in embryos in which Gsk3*β* was disrupted with a translation-blocking MO and a synergistic interaction between Gsk3*β* and *map1b* (data not shown).

To evaluate whether *map1b* disruption results in later developmental defects, *map1b* MO1 and MO2 (10 ng)-injected embryos were imaged at 24 hpf. We observed a disorganization of the hindbrain region in these embryos, characterized by absence of morphological landmarks (Additional file 5: Figure S3) that were more pronounced with MO1 than MO2. Sectioning through the hindbrain of MO1-injected embryos revealed that the disorganization was caused by a partial (*n* = 4 out of 10 embryos) or full (*n* = 1 out of 10 embryos) duplication of the neural tube (Fig. 5b). This striking phenotype was first observed in PCP mutants [12] and is thought to be a consequence of delayed NC [47, 51]. In addition to the disorganized hindbrain, we observed a shortened body axis (Additional file 5: Figure S3), a phenotype often associated with impaired convergent extension in the axial mesoderm [52].

### Truncated Map1b lacking the MT-binding-domain causes delayed NC

In order to confirm the role of Map1b in mediating NC using a MO-independent method, we designed a construct based on a mouse mutation thought to function as a dominant-negative allele [31, 53, 54]. The zebrafish mutant construct, *δmap1b*, encodes the first 571 aa of Map1b, which includes the Map1b light chain (LC1) binding domain (in the heavy chain region) but not the MT-binding domain (6A). Mice that are heterozygous for this mutation have a spectrum of phenotypes including slow growth rates and small eyes, while their homozygous siblings die during embryogenesis [53]. Interestingly, zebrafish embryos injected with *δmap1b* RNA (25, 50, 75 and 100 ng/μl) exhibit an increasingly severe reduction in body and eye size with higher concentrations of RNA (Additional file 6: Figure S4B), suggesting that the truncated Map1b protein functions in a similar manner in both species.

To test whether δMap1b disrupts NC, we injected an intermediate concentration of *δmap1b* RNA (50 ng/μl) and performed the previously described convergence assay. As was reported for *map1b* MO1 and MO2-injected embryos, we observed delays in NC in *δmap1b*-injected relative to controls (Fig. 5a) (uninjected: 272 μm ± 11 μm, *n* = 58 embryos vs δ*map1b*: 355 μm ± 13 μm, *n* = 45 embryos).

The more severe phenotype in *δmap1b*-injected embryos compared to *map1b* MO-injected embryos suggests that δMap1b functions in a dominant-negative manner. Given that δ Map1b retains the ability to bind to LC1 [31], which is implicated in the regulation of MT stability and other MT-independent processes [31, 55], it is likely that depletion of this peptide accounts for some of the pronounced defects observed in injected embryos. In addition, the unbound endogenous heavy chain in *δmap1b*-injected embryos may also play a contributing role.

Together these findings reveal a previously unknown role for Map1b in mediating NC in the neural ectoderm. The fully or partially duplicated neural tube observed in *map1b* MO-injected embryos is consistent impaired NC. Furthermore, the fact that a neural tube, albeit abnormal, forms in Map1b-depleted embryos indicates that Map1b is required for the timely progression rather than completion of neurulation.

### Loss of Map1b results in the loss of stable microtubules

**Fig. 6:**
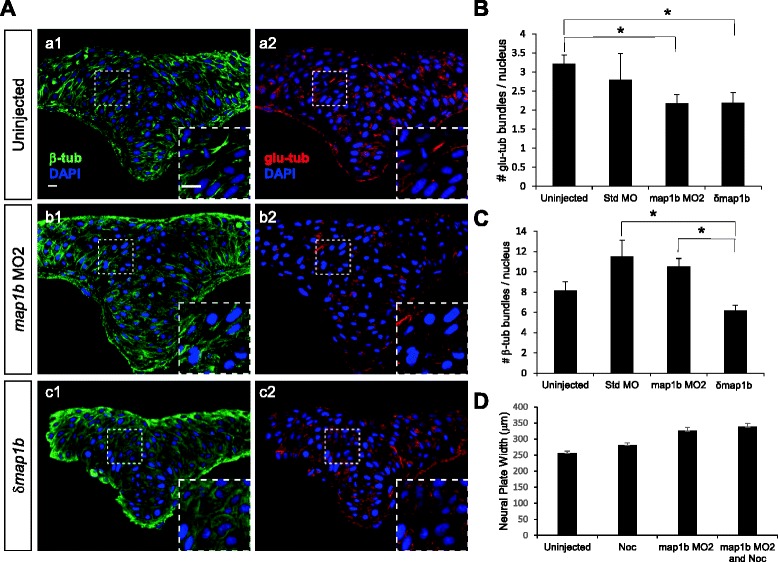
Microtubule stability is altered in Map1b-depleted embryos. **a** Hindbrain sections of uninjected (*a1*, *a2*), *map1b* MO2-injected (*b1*, *b2*) and *δmap1b*-injected (*c1*, *c2*) embryos at 4–5 som immunolabeled with anti-β-tub (green, *a1*, *b1*, *c1*) and anti-glu-tub (red, *a2*, *b2*, *c2*). Nuclei are labeled in blue with DAPI. Insets show higher magnification of boxed areas. Scale bars: 10 μm. **b** Quantification of the average number of glu-tub - labeled bundles per nucleus in uninjected, standard MO-injected, *map1b* MO2-injected and *δmap1b*-injected embryos. (*) Indicates statistical significance (*P* <0.05 for uninjected vs *map1b* MO2 and *P* <0.05 for uninjected vs *δmap1b*-injected) using ANOVA followed by a Bonferroni post test. **c** Quantification of the average number of β-tub labeled bundles per nucleus in uninjected, standard MO-injected, *map1b* MO2-injected and *δmap1b*-injected embryos. (*) Indicates statistical significance (*P* <0.01 for standard MO vs *δmap1b-*injected) using ANOVA followed by a Bonferroni post test (**d**) Quantification of the neural plate width in control (uninjected), *map1b* MO2-injected embryos, nocodazole (3 μΜ)-treated embryos, and *map1b* MO1-injected embryos treated with nocodazole

Previous studies indicate that Map1b maintains a dynamic population of MTs that promotes axonal growth [48, 56]. If Map1b plays a similar role in the neuroectoderm, its loss-of-function should result in increased MT stability. To test this prediction, neural keel (4–5 som) stage uninjected, standard MO-injected, *map1b* MO2-injected and *δmap1b*-injected embryos were sectioned and labeled with anti-β-tub (total MTs), anti-glu-tub (stable, detyrosinated MTs) and DAPI (nuclei) and imaged at the hindbrain level. Unlike the dramatic perturbation of the MT network observed following treatments with nocodazole and paclitaxel (Additional file 2: Figure S2), the overall organization of MTs visualized with β-tub labeling in *map1b* MO2- and *δmap1b-*injected embryos appeared similar to controls (Fig. 6a), although in some samples (insets in Fig. 6a* b1, c1*) the β-tub-labeled bundles were less well defined than those of uninjected (inset in Fig. 6a* a1*) and standard MO (data not shown).

Enhanced MT stability is often manifested in either an increase in MT bundle length or the average number of MT bundles/cell. Quantification of the length of glu-tub- and β-tub-positive bundles in the different treatment groups did not reveal any differences (data not shown). However, the average number of stable, glu-tub-positive bundles per cell (nucleus) revealed an unexpected decrease in *map1b* MO2- and *δmap1b*-injected embryos relative to uninjected embryos (Fig. 6b) (uninjected embryos: 3.2 ± 0.23, *n* = 13 embryos; standard MO-injected embryos: 2.8 ± 0.69, *n* = 3 embryos; *map1b* MO2-injected embryos: 2.18 ± 0.23, *n* = 6 embryos; *δmap1b*-injected embryos: 2.19 ± 0.27, *n* = 9 embryos). Although a decrease in bundle number in experimental groups relative to standard MO-injected embryos was also observed, this number was not statistically significant, most likely due to the smaller sample size of the latter (Fig. 6b). These observations suggest that in the context of NC, Map1b functions to stabilize MTs rather than promote MT dynamics.

A decrease in total bundle number per cell was also observed with β-tub labeling, but only in *δmap1b*-injected embryos (uninjected embryos: 8.16 ± 0.85, *n* = 14 embryos; standard MO-injected embryos: 11.5 ± 1.6, *n* = 5 embryos; *δmap1b*-injected embryos: 6.18 ± 0.54, *n* = 9 embryos) (Fig. 6c). This reduction is unlikely to reflect the loss of stable MTs from the total MT population given that a decrease in total MTs was not observed in *map1b* MO2-injected embryos. Thus, δMap1b may disrupt total MTs (dynamic and stable) whereas *map1b*-MO2 alters stable MTs specifically. However, a more likely explanation, is that the apparent loss of total MT bundles in *δmap1b*-injected embryos reflects subtle changes in the organization of the MT cytoskeleton (bundles that are less well defined) that make the automated quantification method less accurate.

To more directly tease apart the role of Map1b in regulating stable versus dynamic MTs, we also analyzed the levels and distribution of tyrosinated α-tub (dynamic MTs) in *map1b*-MO2- and *δmap1b*-injected embryos. We did not observed an obvious difference in the number and organization of tyr-tub MTs in these embryos (*map1b* MO2-injected embryos: *n*= 4 embryos; *δmap1b*-injected embryos: *n*= 3 embryos) relative to controls (uninjected embryos: *n* = 5 embryos). We did however notice an increase in the number of puncta labeled with anti tyr-tub (arrowheads in Additional File 7: Figure S5). Since re-tyrosination has been reported to occur on the non-assembled tubulin dimer pool [57], these puncta may correspond to depolimerized tubulin. Overall these observations suggest that loss of Map1b does not impact dynamic MTs.

To further test whether Map1b is implicated in MT stabilization, we performed a nocodazole sensitization test in *map1b* MO2-injected (4 ng) embryos. However, treatment of these embryos with a low dose of nocodazole (1 μg/ml) did not worsen NC defects (Fig. 5d). This may be due to intrinsic differences in the mechanisms by which these molecules alter MT properties. Alternatively, Map1b may function via additional MT-independent mechanisms to promote NC.

### Map1b functions cell-autonomously to regulate cell elongation

**Fig. 7:**
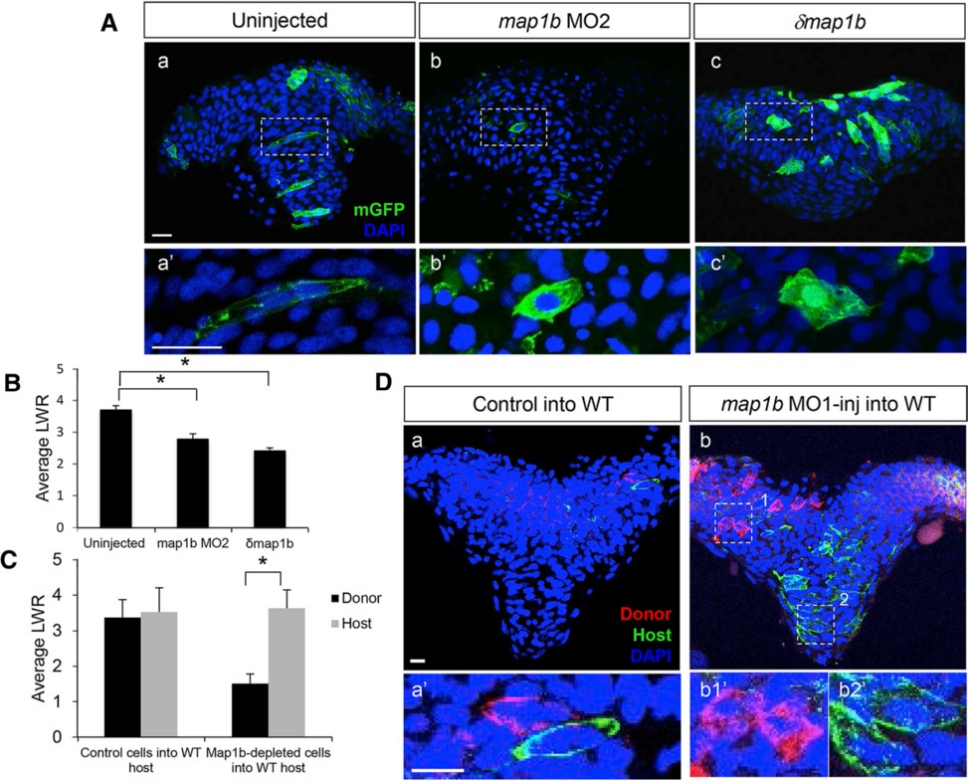
Map1b functions cell autonomously in the neural ectoderm. **a** Hindbrain sections of 4–5 som uninjected (a, a’), *map1b* MO2-injected (b, b’) and *δmap1b*-injected (c, c’) embryos mosaically-expressing mGFP (green). Nuclei are labeled in blue with DAPI. (a’–c’) Higher magnification of boxed areas in (a–c) respectively. Scale bars: 20 μm. **b** Quantification of the LWR of cells in 4–5 som uninjected, *map1b* MO2-injected and *δmap1b*-injected embryos. (*) Indicates statistical significance (*P* <0.01 for uninjected vs *map1b* MO2 and *P* <0.001 for uninjected vs *δmap1b*-injected) using a Kruskal-Wallis test followed by Dunn’s post-hoc test. **c** Quantification of the LWR of control donor cells vs host WT cells and *map1b* MO1-injected donor cells vs host WT cells. (*) Indicates statistical significance (*P* <0.0001) using Student’s *T*-test. **d** Hindbrain sections of 4–5 som WT hosts mosaically-expressing mGFP and transplanted with (a) mRFP-labeled control donor cells or (b) mRFP-labeled *map1b*-MO1 donor cells. Nuclei are labeled with DAPI (blue). (a’, b1’ and b2’) Higher magnifications of boxed areas in (a, b1 and b2) respectively. Scale bars: 10 μm

In order to identify the cellular mechanisms underlying delayed NC in Map1b-deficient embryos, we analyzed the morphology of hindbrain cells mosaically expressing mGFP at the neural keel (4–5 som) stage. We observed that in contrast to control (uninjected) cells (Fig 6a* a–a’*), cells in *map1b* MO1 (data not shown), *map1b* MO2- and *δmap1b*-injected embryos failed to elongate (Fig. 7a* b – b’, c-c'*), as was observed with drug-treated embryos (Fig. 3c, d). LWR measurements of control and Map1b*-*deficient cells revealed a significant difference in cell shape (LWR of uninjected cells = 3.70 ± 0.1, *n* = 111 cells from 7 embryos; LWR of cells from embryos injected with *map1b* MO2 = 2.78 ± 0.2, *n* = 43 cells from 5 embryos; LWR of cells from embryos injected with δ*map1b* = 2.41 ± 0.1, *n* = 135 cells from 9 embryos) (Fig. 7b). Thus, Map1b is required for cell elongation during NC and its ability to regulate MTs may underlie this process.

Since *map1b* is expressed in the mesoderm and neuroectoderm (Fig. 4), it is possible that the widened neural plate of Map1b-deficient embryos is an indirect consequence of defective convergent extension movements in the mesoderm [52]. If Map1b functions in a cell-autonomous manner in the neuroectoderm to regulate cell elongation, then Map1b-deficient cells isochronically transplanted into WT hosts are expected to be rounded. Conversely, isochronic transplantation of control (WT) cells into Map1b-deficient embryos should not impact the ability of these donor cells to adopt their correct elongated morphology.

To perform the first isochronic transplantation experiment, donor embryos were injected with RNA encoding mRFP with or without *map1b* MO1 (10 ng) and host embryos were injected with *mGFP* DNA (which is mosaically expressed). Cell shapes of both donor (red) and host (green) cells were analyzed in hindbrain sections of host embryos fixed at the neural keel stage (4–5 som). We observed that transplanted control cells were similar in shape to WT host cells (LWR of control cells = 3.37 ± 0.5, *n* = 100 cells from 18 embryos; LWR of host cells = 3.52 ± 0.69, *n* = 88 cells from 14 embryos; Fig. 7c, d* a–a’*), whereas transplanted Map1b-deficient cells consistently appeared rounder than WT host cells (LWR of *map1b* MO1-injected cells = 1.51 ± 0.27, *n* = 135 cells from 14 embryos; LWR of WT host cells = 3.63 ± 0.52, *n* = 92 cells from 14 embryos; Fig. 7c, d* b–b.2’*). There was no apparent bias to the location of transplanted cells in the neural tube of their hosts, eliminating position as a contributing factor to differences in cell shape. These data reveal that Map1b functions cell autonomously to regulate cell elongation in the neural ectoderm.

The reciprocal isochronic transplantation could not be completed as the Map1b-deficient hosts did not survive the transplantation.

### Map1b is required for polarized migration during NC

**Fig. 8:**
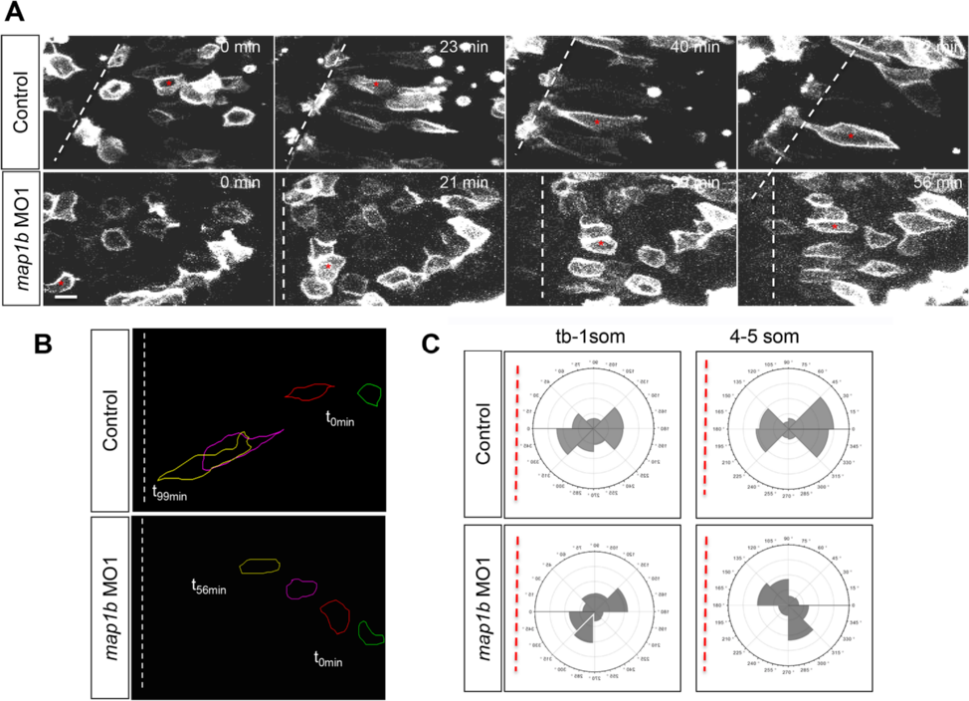
Polarized migration is disrupted in Map1b-depleted embryos. **a** Selected frames from time-lapse imaging of control (uninjected) and *map1b*-depleted mGFP-expressing cells in the neural plate. The white dotted line indicates the dorsal midline, when visible in the imaging field. Time elapsed (minutes) is indicated in the upper right corner. Red asterisks indicate individual cells identified in multiple frames. Scale bar: 10 μm. **b** Representative traces of control and *map1b*-depleted cells traced over time. Traces corresponding to time 0 (t0 min, green) are to the right and traces of older cells (t56 min and higher, yellow) are to the left. **c** Plot of the average distribution of membrane protrusions in representative mGFP- labeled control and *map1b*-depleted cells at the neural plate stage. The red dotted line represents the position of the dorsal midline

To test whether Map1b plays a role in polarized cell migration during NC, mGFP-labeled cells in control (uninjected) and *map1b* MO1(10 ng)-injected embryos were imaged using time-lapse microscopy (Fig. 8a and Additional files 8 and 9). Cell tracing revealed that control cells (from uninjected embryos) were initially rounded and gradually elongated as they approached the midline. In contrast, cells in *map1b* MO1-injected embryos took longer to elongate, consistent with the LWR measurements of cells in fixed preparations. In addition, their migration towards the midline was delayed (Fig. 8b; *n* = 3 embryos; 6–9 cells/embryo).

In order to determine whether delayed migration in *map1b* MO1-injected embryos was caused by defective protrusive activity, as observed in drug-treated embryos, the angular distribution of plasma membrane extensions was quantified and ploted. At the neural plate (tb-1 som) and neural keel (4–5 som) stages, membrane protrusions of control cells were biased towards the medio-lateral axis, whereas the membrane protrusions in Map1b-deficient cells were less polarized and failed to align with the medio-lateral axis (Fig. 8c). Together these findings suggest that Map1b mediates both cell elongation and the polarized orientation of protrusive activity, two cell behaviors that are MT-dependent.

## Discussion

### MTs are required for NC

Cellular dynamics during convergent extension movements in vertebrates are powered by actin polymerization, cell-cell adhesion and cell-extracellular matrix (ECM) interactions [52]. Since MTs play a prominent role in cell migration [17, 22], it seems intuitive that they would also be implicated in the mechanics of cellular rearrangements during convergent extension in the mesoderm and neurectoderm. There is some experimental evidence supporting MT-mediated cellular rearrangement in the mesoderm, however, there is a dearth of data on the involvement of MTs during NC. With respect to the mesoderm, disruption with nocodazole in early gastrula *Xenopus* embryos prevents mediolateral intercalation, involution and convergent extension of the marginal zone (the precursor of the mesoderm) [58]. Kwan and Kirschner further demonstrated that treatment of *Xenopus* dorsal marginal zone explants with nocodazole but not taxol prevents lamellipodia formation, indicating that the bulk of polymerized tubulin rather than MT dynamics (which would be altered by both drug treatments) is important for convergent extension [59]. In zebrafish embryos, MTs are known to mediate cell-cell contacts and initiation of planar polarity, by localizing PCP pathway component Prickle in a polarized manner during mesodermal convergent extension [60].

In amniotes, narrowing of the neural plate is brought about by a combination of cell elongation and intercalation. In chick embryos, treatment of the neural plate with nocodazole prevents cell lengthening along the apico-basal axis, resulting in a wider neural plate [61]. While it is currently unknown whether MTs also power cell intercalation in amniotes, the medio-lateral oriented basal protrusions that drive cellular rearrangement in the mouse epithelialized neural plate [9] are reminiscent of MT-dependent polarized basal protrusions in *C. elegans* epithelial cells undergoing dorsal closure [62], raising the possibility that MT-based mechanisms may also be employed to narrow the neural in chick and mouse embryos.

We have previously shown that MTs in neural plate cells have a radial organization, which is characteristic of migratory cells. Following NC, MTs become linear, an architecture often observed in epithelial cells [42]. We report here that the levels of stable MTs steadily increase as neurulation proceeds. Our functional analysis using MT-disrupting drugs further suggests that proper regulation of MT stability is essential for both cell elongation and polarized migration during NC. In addition, the fact that protrusive activity is still observed (albeit random) in nocodazole-treated embryos, indicates that MTs are required for polarization but not the formation of these membrane extensions.

Thus, despite the more prominent mesenchymal properties of zebrafish neural plate cells relative to their amniote counterparts, evidence suggests that MTs and their regulators play a central role in driving NC in vertebrates.

### Map1b promotes stable microtubules

The prominent changes in MT stability during neurulation are likely to be regulated by microtubule associated proteins. We show here that stable/detyrosinated MTs are lost in Map1b-deficient embryos. The apparent selective reduction in detyrosinated MTs (observed in *map1b* MO2-injected embryos) argues against a role for Map1b as a general MT stabilizing factor. Rather, Map1b may protect stable MTs or promote the α-tub detyrosination event that is revealed by glu-tub labeling.

Despite the focus on MTs in this study, it is likely that Map1b also influences the actin cytoskeleton. In this regard, Map1b is known to bind actin in addition to MTs [25, 63], thereby crosslinking the two cytoskeletons. Furthermore, Rac1 and Cdc42 are downstream effectors of Map1b [64] that are both implicated in the crosstalk between actin and MTs [65].

### Map1b regulates distinct cell behaviors during NC

We have previously shown that, during NC, cells elongate as they migrate towards the dorsal midline. These cellular dynamics are accompanied by extensive protrusive activity polarized along the medio-lateral axis [8]. Since perturbation of MT dynamics with nocodazole or paclitaxel prevents cell elongation and polarized migration in the zebrafish neural plate (this study) and other contexts [23, 66-71], we investigated whether these cell behaviors are also altered in Map1b-depleted embryos.

We found that despite the broad distribution of mRNA in mesodermal and ectodermal cells, Map1b is required cell autonomously for cell elongation in the neural tissue. A recent study has shown that a polarized population of dynamic MTs is required for cell length maintenance in the zebrafish neural tube [23]. While our studies reveal a role for Map1b in promoting stable MTs rather than MT dynamicity, it is likely that both MT populations contribute to cell elongation.

In addition to cell elongation, directional migration is also defective in Map1b-depleted embryos. Impaired migration may also be attributable to abnormal MTs, as stable MTs, anchored at the cell cortex, are thought to function as tracks to deliver regulators of actin polymerization to the leading edge [17]. Whether the same population of Map1b-regulated MTs mediates cell elongation and migration in the zebrafish neural tube is unclear.

Analysis of protrusive activity in Map1b-depleted embryos revealed a lack of biased orientation along the medio-lateral axis and ectopic persistent protrusions on the anterior and posterior pole of neural cells. This abnormal protrusive activity may underlie the delay in cell elongation and migration. It is unclear how Map1b biases protrusive activity medially. However, the recent finding that Map1b binds and sequesters EB3 in the cytosol of developing neuronal cells [72] raises an interesting possibility. EB3 is a MT-plus end binding protein that is enriched in growth cones and has been shown to coordinate the interaction between F-actin (required for protrusive activity) and dynamic MTs during neuritogenesis [73]. Furthermore, EB3-capped MT plus ends orient towards the leading edge in migrating cells, possibly in response to an extracellular signal [17]. In this context, Map1b may regulate polarized protrusive activity by controlling the levels of EB3 available to associate with MT plus ends. In the absence of Map1b, increased binding of EB3 to MTs plus ends could cause the formation of ectopic F-actin nucleation.

Despite the significant increase in neural plate width in *map1b* MO-injected embryos, the neural tube eventually forms (albeit abnormally), indicative of a delay rather than blockage of NC. A similar outcome was also observed following depletion of PCP pathway components, suggesting compensatory mechanisms that ensure proper completion of neural development.

Whether *map1b* function during NC is conserved remains to be determined, as neural tube defects have not been reported in mouse *map1b* knockouts [74], possibly due to functional redundancy among MAP family members [75, 76] or distinct cellular mechanisms underlying the narrowing of the neural plate. Despite these differences, loss of Map1b function in mice also causes a delay rather than a blockage in neural development [34].

## Conclusions

We show that MTs become progressively more stable as neurulation progresses. Drug treatments that either destabilize or hyperstabilize MTs impair NC by disrupting cell elongation and polarization, indicating that the regulation of MT stability is a key event during neural tube development. We demonstrate that the microtubule-associated protein Map1b is broadly expressed during neurulation and promotes stable MTs. Furthermore, loss of Map1b function causes a delay in NC, cell autonomous disruption of cell elongation, impaired directional migration and polarized protrusive activity. Based on these findings, we propose that Map1b enables NC at least in part by maintaining a population of stable MTs.

Collectively, these studies identify *map1b* as a key regulator of early morphogenetic movements in the neural tube. It will be interesting in the future to identify the signaling pathways that function upstream of Map1b to control the MT cytoskeleton during NC.

## Methods

### Zebrafish strains

Studies were performed using wildtype (AB) strains. All experiments were approved by the University of Maryland, Baltimore County’s Institutional Animal Care and Use Committee (IACUC) and were performed according to national regulatory standards.

### Embryo staging

Staging was done according to [77]. Stages of neurulation were defined as previously described [8].

### Cloning of zebrafish *map1b*

RNA was extracted from 24 hpf AB embryos using TRIzol (Invitrogen, cat no. 15596–026). cDNA was synthesized with RETROscript (Invitrogen, cat no. AM1710) and oligodT primers. Primers were designed to amplify a conserved, 302 bp region of zebrafish *map1b* corresponding to exon 5 (accession # XM_003198629):

- Forward primer: 5’-AGCACCGTACATCCAGCCAACA-3’
- Reverse primer: 5’-GCAAACAATGCAGAGTCACCCCGT-3’

PCR was performed using PfuUltra (Agilent Technologies, cat no. 600385) and products were cloned into PCR II-TOPO vector (Invitrogen, cat no. K4600-01).

The *δmap1b* construct, a codon optimized sequence encoding the first 571 aa of zebrafish Map1b, was synthesized by Genewiz based on the published zebrafish *map1b* sequence (accession # XM_003198629). *δmap1b* was subsequently subcloned into the pCS2+ vector.

### Nucleic acid and morpholino injections

DNA encoding membrane-targeted Green Fluorescent Protein (mGFP) (Richard Harland, University of California, Berkeley, CA, USA) and Red Fluorescent Protein (mRFP) [78] for mosaic expression were prepared using a midiprep kit (Macherey-Nagel, cat. no. 740410.10) and injected (50–200 pg) into one- to eight-cell stage embryos.

For RNA injections, *mGFP or mRFP* expressing plasmids were linearized with NotI and transcribed using SP6 mMESSAGE mMACHINE kit (Ambion, cat. no. AM1340). 50 pg of RNA was injected into one- to four-cell stage embryos.

MOs were synthesized by GeneTools (Philomath, Oregon, USA) and injected into one- to four-cell stage embryos: *map1b* splice-blocking MO1 (4 or 10 ng), *map1b* splice-blocking MO2 (4 or 10 ng), *map1b* translation (ATG)-blocking MO3 (10 ng) and, as negative controls, *pard3* (10 ng) and a standard negative control MO recommended by GeneTools that targets a human beta-globin intron, causing little change in phenotype in any known test system (10 ng).

- *map1b* MO1: 5’-CCAAGAAAAACAGTCACTTACCTCT- 3’
- *map1b* MO2: 5’-AATTTGACTTACAGATTGGAGAGCT- 3’
- *map1b* MO3: 5’-CCGCAGTATCAACCAGCGTCGCCAT- 3’
- *pard3* MO: 5’ TCAAAGGCTCCCGTGCTCTGGTGTC 3’ [79]
- *Gsk3β* MO: 5’-GTTCTGGGCCGACCGGACATTTTTC-3’ [80]
- Standard MO: 5’-CCTCTTACCTCAGTTACAATTTATA- 3’ [81]

Microinjections were performed using a PCI-100 microinjector (Harvard Apparatus, Holliston, MA, USA).

### Cell transplantation

Transplantation was performed as described in [82]. 50–100 cells from donors were transplanted isochronically into the animal pole of host embryos at the sphere to dome stage.

### Drug treatments

Early neural keel stage (2–3 som) embryos mosaically expressing mGFP were manually dechorionated and exposed to nocodazole (concentrations ranging from 3 to 32 μM) (Sigma, cat. no. M1404) or paclitaxel (50 μM) (Sigma, cat. no. T7191) until embryos reached 4–5 som stage (~30 min) at 28 °C. The embryos were immediately fixed overnight in 4 % paraformaldehyde (PFA) diluted in PBS at 4°C.

### Time-lapse confocal microscopy

Time-lapse microscopy was performed as previously described [83]. Embryos were imaged using a Leica confocal microscope (Leica SP5 TCS 4D) at 30 s-1 min intervals. Images were analyzed using the Leica LAS software, Image J (NIH) and Adobe PhotoShop.

### Labeling and imaging of fixed preparations

For immunolabeling, embryos were fixed for 3 h with 4 % PFA diluted in MAB buffer (80mM KPIPES, 5mM EGTA, 1mM MgCl2, 0.2 % Triton-X, pH 6.4) at room temperature. Embryos were then sectioned (40μm, 1500 Sectioning System) and immunolabeling on floating sections was carried out as in [84].

Antibodies used: mouse anti-β-tubulin (Sigma, Clone: TUB 2.1) at 1:500; rabbit anti-α-tubulin (Genetex, Clone: GTX108784) at 1:500; rabbit anti-tyrosinated tubulin (Millipore, ABT171) at 1:1000; rabbit anti-glu-tubulin (Millipore, Clone: AB201) at 1:1000 and rabbit anti-GFP (Invitrogen, cat. no. A11122) at 1:1000. Secondary antibodies conjugated to Alexa 488, Alexa 594, or Cy3 (Molecular Probes, cat. nos A11001 and A11008; Molecular Probes cat. A21442; Invitrogen cat. no. A10520) were used at a 1:500 dilution. Alexa Fluor 488-conjugated Phalloidin (Invitrogen, cat. no. A12379) at 1:75 and DAPI (Invitrogen, cat. No. D1306) were used according to manufacturer’s instructions.

For cell shape analysis, *mGFP* or *mRFP* RNA/DNA injected embryos were sectioned and either imaged directly (mRFP) or immunolabeled with anti-GFP prior to imaging. All fluorescently labeled sections were imaged using an SP5 confocal microscope (Leica SP5 TCS 4D).

### Wholemount *in situ* hybridization

ISH was conducted as described [85]. Plasmid containing *dlx3* (obtained from Igor Dawid, NIH) was linearized with *NotI* and T7 polymerase was used to generate anti-sense probe. *map1b* (in pCRII-TOPO vector) was linearized using *NotI* and *KpnI* for sense and anti-sense probes, respectively. T7 and SP6 polymerases were used to generate anti-sense and sense probes respectively. ISH labeled embryos were sectioned and imaged using a Zeiss Axioscope2 microscope.

### Whole cell lysis and Western blotting

~200 embryos were collected, dechorionated, and batch deyolked as described elsewhere [86] with the following modifications. After deyolking, cells were vortexed for one minute in lysis buffer (100 mM PIPES, 0.5 % Nonidet P-40, 1 mM MgCl2, 2 mM EDTA, 1 mM dithiotheitrol, 1:100 dilution of Sigma protease inhibitor cocktail, cat. No. P8340) and centrifuged for 5 min at high speed. Supernatant was boiled for 5 min in 2x SDS-loading buffer, run on a 4-20 % Tris-glycine polyacrylamide gel (BioRad, cat. no. 456–1085) and transferred onto a nitrocellulose membrane (Pall Corporation, cat. no. S80209). Blots were blocked in 5 % dry milk dissolved in PBST (1X PBS, 0.5 % Tween) for 30 min, then incubated with 1:1000 anti-α-tub (Millipore, clone DM1A) or 1:500 anti-glu-tub (Millipore, Clone: AB201). Blots were then incubated with 1:1000 anti-mouse or anti-rabbit HRP-conjugated secondary antibodies (Santa Cruz, cat. nos. SC-2005 and SC-2004). Blots were developed using an enhanced chemiluminescence kit (Thermo Scientific, cat. no. 34079).

### Measurements and statistical analysis

#### Length-to-Width ratios

LWRs were calculated as previously described [8].

#### Tracing and quantification of cell behaviors

Cell tracing of single cells over multiple time frames was performed using Metamorph (MolecularDevices).

#### Protrusion analysis

Stacks of images from time-lapse microscopy were flattened to a single frame per time point using Volocity v5.5 (Perkin-Elmer) and exported as tifs. Interphase cells that stayed in frame for the extent of the movie were selected. Using a plugin created for imageJ (NIH), individual cells were threshholded and the outline traced. Each cell was then divided into 8 segments based on the centroid and the orientation to the midline. Finally individual frames were overlaid and any new membrane extensions were counted for each frame. The percent protrusions for each section was then calculated and plotted in Mathematica v9 (Wolfram).

#### Synteny analysis

Performed using synteny (http://cinteny.cchmc.org/).

#### Statistical analysis

InStat (GraphPad) was used to run statistical analysis on data sets. Student’s *T*-test, ANOVA followed by Bonferroni or Kruskal-Wallis test followed by Dunn’s post-hoc test were used to analyze data groups as appropriate.

#### Quantification of Western band intensity

Band intensity was determined using digital scans followed by analysis with ImageJ (NIH). The area used to measure was constant for all experiments.

#### Quantification of MTs

Measurements of MT bundle length and number of MT bundles/nuclei were done using Volocity (Perkin-Elmer). A maximum intensity projection of the total confocal sections (40 μm) imaged using the Leica SP5 was generated. The neural keel was cropped to quantify only MT signal within the neural tissue. Filters that identified nuclei (DAPI), stable MTs (glu-tub) and total MTs (β-tub) based on the standard deviation of intensity was used. To compensate for objects close together, an automated algorithm that separates object was run as part of the filter. The measurements were automatically collected and analyzed in excel

## Additional files

Additional file 1: Figure S1. Microtubule stability increases during neurulation. (A) Western blot of whole cell lysates blotted for glu-tub (stable MTs) and α-tub (total MTs) at neural plate (tb-1 som), neural keel (4–5 som) and neural rod (12 som) stages. Two bands (1) and (2) are observed for glu-tub. (B) Ratio of stable:total MTs at neural plate (tb-1 som), neural keel (4–5 som) and neural rod (12 som) stages, calculated using glu-tub bands 1 and 2 (A). (PDF 83 kb) Additional file 2: Figure S2. Nocodazole and paclitaxol disrupt microtubule organization. (a-c’) Hindbrain sections of 4–5 som control (a, a’), nocodazole-treated (b, b’) and paclitaxel-treated (c, c’) embryos immunolabeled with anti-β-tub. (a’-c’) Higher magnifications of boxed areas in panels (a,b and c) respectively. Scale bars: 20 μm. (PDF 8922 kb) Additional file 3:
**Time lapse imaging of cell behaviors during NC in a WT embryo (high magnification).** Time-lapse movie (1 min intervals) of a control, mGFP-labeled embryo imaged from a dorsal view, anterior towards the top, beginning approximately at the 2–3 som stage and extending to the 6–7 som stage. (MOV 48 kb) Additional file 4:
**Time lapse imaging of cell behaviors during NC in a nocodazole-treated embryo (high magnification).** Time-lapse movie (1 min intervals) of a nocodazole-treated embryo, mosaically expressing mGFP, imaged from a dorsal view, anterior towards the top, beginning approximately at the 2–3 som stage. (MOV 163 kb) Additional file 5: Figure S3. Efficacy of *map1b* MOs. (A) Schematic representation of zebrafish *map1b*, showing *map1b* MO1 binding site at the exon 4- intron 4 splice junction (red line and lettering), *map1b* MO2 binding site at the intron 4- exon 5 splice junction (blue line and lettering) and *map1b* MO3 at the translational start site (green line and lettering). Exons are represented by black boxes with corresponding numbers on top. (B) RT-PCR analysis of the region targeted by splice MOs . The upper (750bp) and lower (300bp) bands correspond to unspliced and spliced product respectively. (C) Quantification of the width of the neural plate (tb- 1 som) of control embryos and embryos injected with *map1b* MO1 (4 ng), *map1b* MO3 (10 ng) and *map1b* MO1 (4 ng) + *map1b* MO3 (10 ng). * Indicates statistical significance using a Kruskal-Wallis test followed by Dunn’s post-hoc test (*P* <0.05 compared to the rest of the groups). (D) Side views of 24 hpf uninjected, *map1b* MO1-injected (10 ng) and *map1b* MO2-injected (10 ng) embryos. Black line indicates morphological defects in the hindbrain region. Anterior is to the left, dorsal is up. Scale bar: 250 μm. (PDF 4983 kb) Additional file 6: Figure S4. δmap1b construct and RNA titration. Representation of zebrafish full length Map1b and δMap1b protein indicating percent amino acid similarity to human ortholog. Black represents the highest level of homology and white, the lowest. Hatch marks indicate the MT-binding domain (MTBD). (B) Titration analysis of *δmap1b* RNA. Percent of embryos with WT, mild, moderate, severe and very severe phenotypes are indicated in the table for each concentration of RNA. (C) 24 hpf embryos correspond to the phenotypic categories in the table (a: WT, b: mild, c: moderate, d: severe e: very severe). Black arrowhead indicates missing eye. (PDF 2953 kb) Additional file 7: Figure S5. Dynamic microtubules appear normal in Map1b-depleted embryos. Hindbrain sections of embryos at the neural keel (4–5 som) stage immunolabeled with anti-tyr-tub (dynamic MTs) in red (a2, b2, c2) anti-β-tub (total MTs) in green (a3, b3, c3). (a1, b1, c1) Red-Green overlay (yellow) with nuclei labeled in blue using DAPI. Boxed areas are shown in higher magnification in (a2-c3). Arrowheads indicate puncta exclusively labeled with anti-tyr-tub. Scale bars: 10 μm. (PDF 9616 kb) Additional file 8:
**Time lapse imaging of NC in a WT embryo.** Time-lapse movie (1 min intervals) of a control, mGFP-labeled embryo imaged from a dorsal view, anterior towards the top, beginning approximately at the 2–3 som stage and extending to the 6–7 som stage.] (MOV 581 kb) Additional file 9:
**Time lapse imaging of NC in a** ***map1b*** **MO1-injected embryo.** Time-lapse movie (1 min intervals) of a *map1b* MO1 (10 ng)-injected embryo mosaically expressing mGFP, imaged from a dorsal view, anterior towards the top, beginning approximately at the 2–3 som stage and extending to the 6–7 som stage. (MOV 538 kb)

## Abbreviations

Hpf: Hours-post-fertilization
Map1b: Microtubule associated protein 1 b
MT: Microtubule
MO: Morpholino
NC: Neural convergence
Som: Somites
