# Supplementary figures and images for: Microtubule-associated protein 1b is required for shaping the neural tube
Source: Neural Dev. 2016 Jan 18;11:1. doi: 10.1186/s13064-015-0056-4 (PMC4717579; doi:10.1186/s13064-015-0056-4)

**A**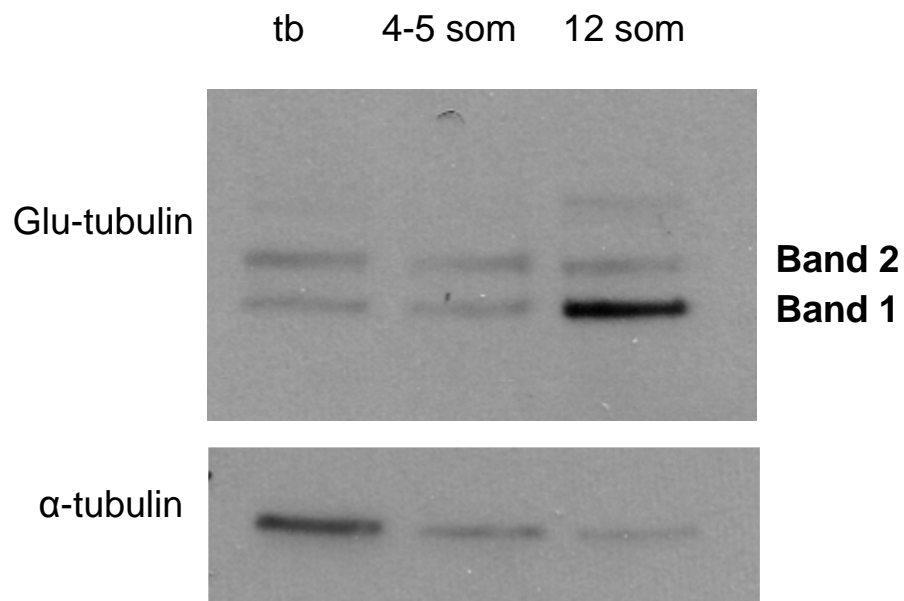**B**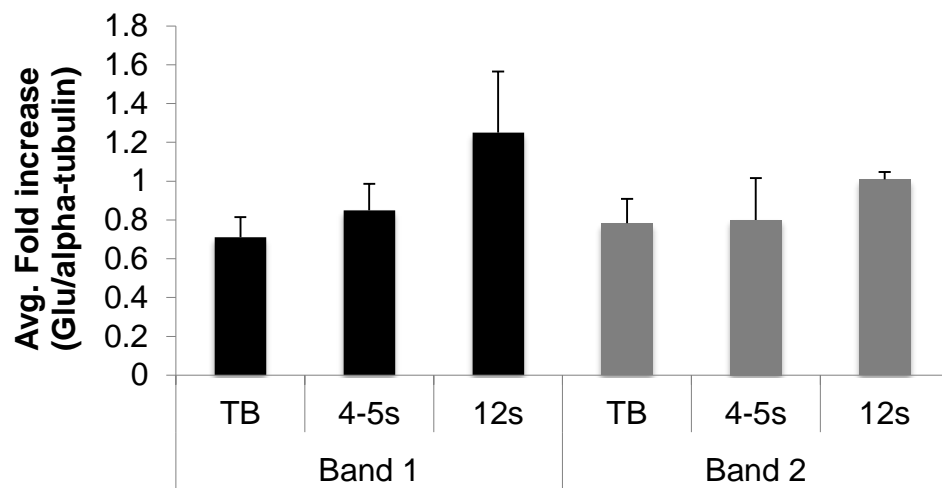

Supplement: Additional file 1: Figure S1. — Microtubule stability increases during neurulation. (A) Western blot of whole cell lysates blotted for glu-tub (stable MTs) and α-tub (total MTs) at neural plate (tb-1 som), neural keel (4–5 som) and neural rod (12 som) stages. Two bands (1) and (2) are observed for glu-tub. (B) Ratio of stable:total MTs at neural plate (tb-1 som), neural keel (4–5 som) and neural rod (12 som) stages, calculated using glu-tub bands 1 and 2 (A). (PDF 83 kb) [file 13064_2015_56_MOESM1_ESM.pdf]

Control

Nocodazole-treated

Paclitaxel-treated

a

b

c

$\beta$ -tub  
DAPI

a'

b'

c'

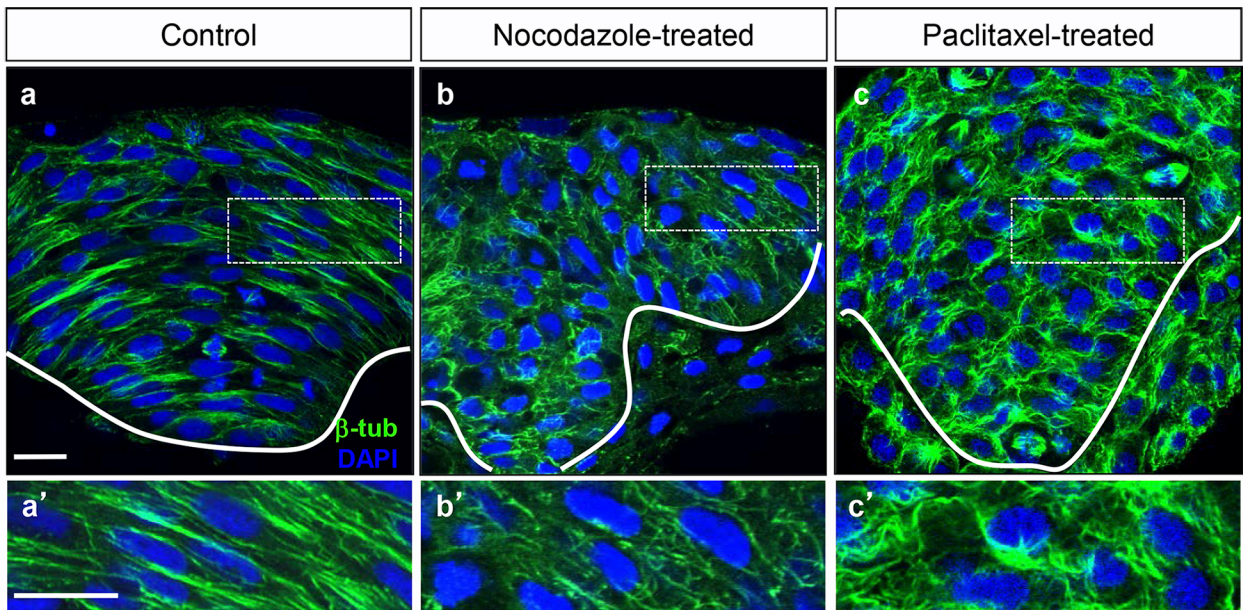

Supplement: Additional file 2: Figure S2. — Nocodazole and paclitaxol disrupt microtubule organization. (a-c’) Hindbrain sections of 4–5 som control (a, a’), nocodazole-treated (b, b’) and paclitaxel-treated (c, c’) embryos immunolabeled with anti-β-tub. (a’-c’) Higher magnifications of boxed areas in panels (a,b and c) respectively. Scale bars: 20 μm. (PDF 8922 kb) [file 13064_2015_56_MOESM2_ESM.pdf]

**A**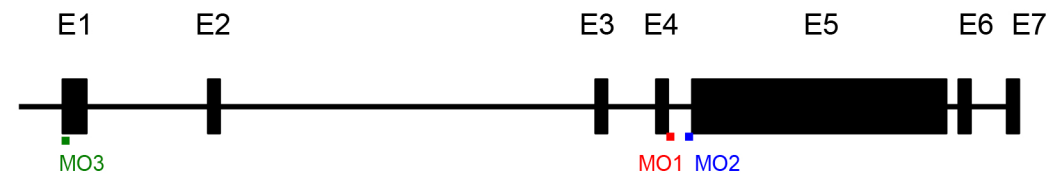**B**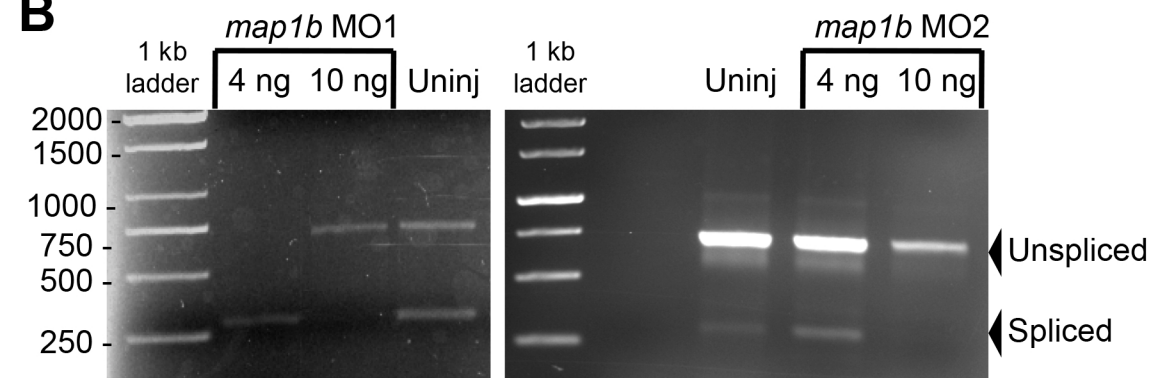**C**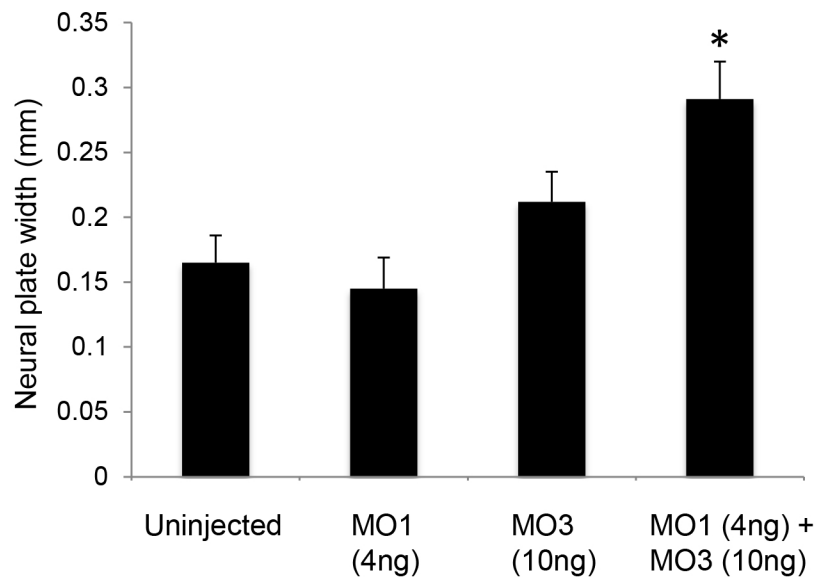**D**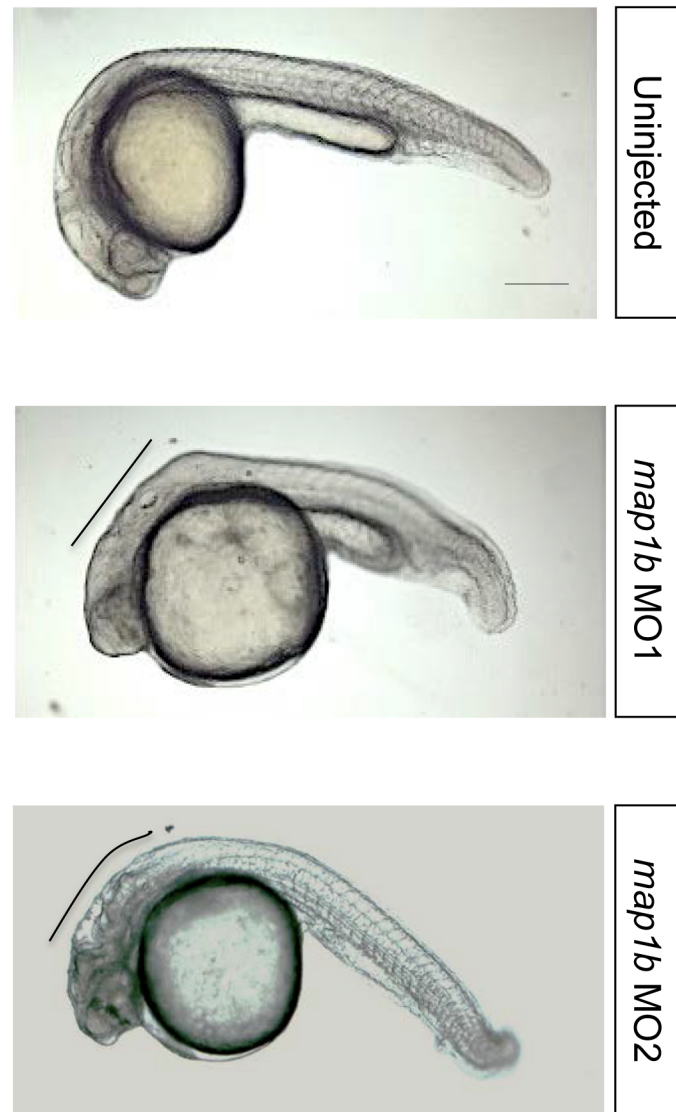

Supplement: Additional file 5: Figure S3. — Efficacy of map1b MOs. (A) Schematic representation of zebrafish map1b, showing map1b MO1 binding site at the exon 4- intron 4 splice junction (red line and lettering), map1b MO2 binding site at the intron 4- exon 5 splice junction (blue line and lettering) and map1b MO3 at the translational start site (green line and lettering). Exons are represented by black boxes with corresponding numbers on top. (B) RT-PCR analysis of the region targeted by splice MOs . The upper (750bp) and lower (300bp) bands correspond to unspliced and spliced product respectively. (C) Quantification of the width of the neural plate (tb- 1 som) of control embryos and embryos injected with map1b MO1 (4 ng), map1b MO3 (10 ng) and map1b MO1 (4 ng) + map1b MO3 (10 ng). * Indicates statistical significance using a Kruskal-Wallis test followed by Dunn’s post-hoc test (P <0.05 compared to the rest of the groups). (D) Side views of 24 hpf uninjected, map1b MO1-injected (10 ng) and map1b MO2-injected (10 ng) embryos. Black line indicates morphological defects in the hindbrain region. Anterior is to the left, dorsal is up. Scale bar: 250 μm. (PDF 4983 kb) [file 13064_2015_56_MOESM5_ESM.pdf]

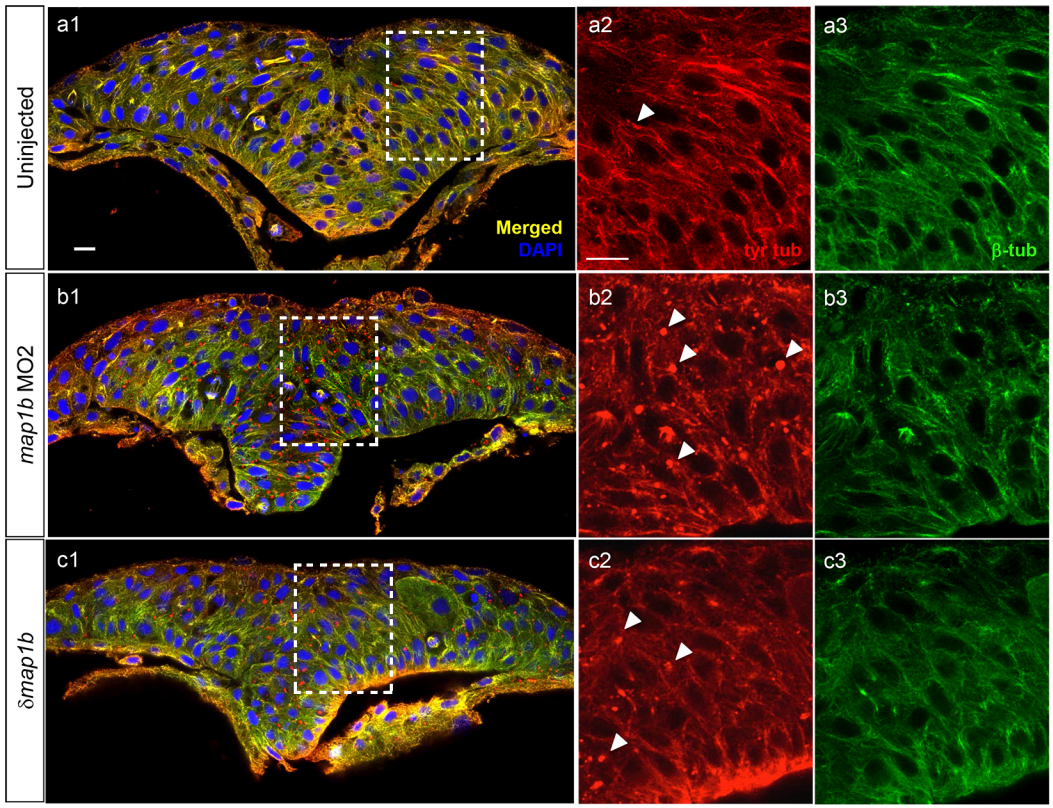

Supplement: Additional file 7: Figure S5. — Dynamic microtubules appear normal in Map1b-depleted embryos. Hindbrain sections of embryos at the neural keel (4–5 som) stage immunolabeled with anti-tyr-tub (dynamic MTs) in red (a2, b2, c2) anti-β-tub (total MTs) in green (a3, b3, c3). (a1, b1, c1) Red-Green overlay (yellow) with nuclei labeled in blue using DAPI. Boxed areas are shown in higher magnification in (a2-c3). Arrowheads indicate puncta exclusively labeled with anti-tyr-tub. Scale bars: 10 μm. (PDF 9616 kb) [file 13064_2015_56_MOESM7_ESM.pdf]
