# Supplementary material for: Microtubule-associated protein 1b is required for shaping the neural tube
Source: Neural Dev. 2016 Jan 18;11:1. doi: 10.1186/s13064-015-0056-4 (PMC4717579; doi:10.1186/s13064-015-0056-4)

**A**

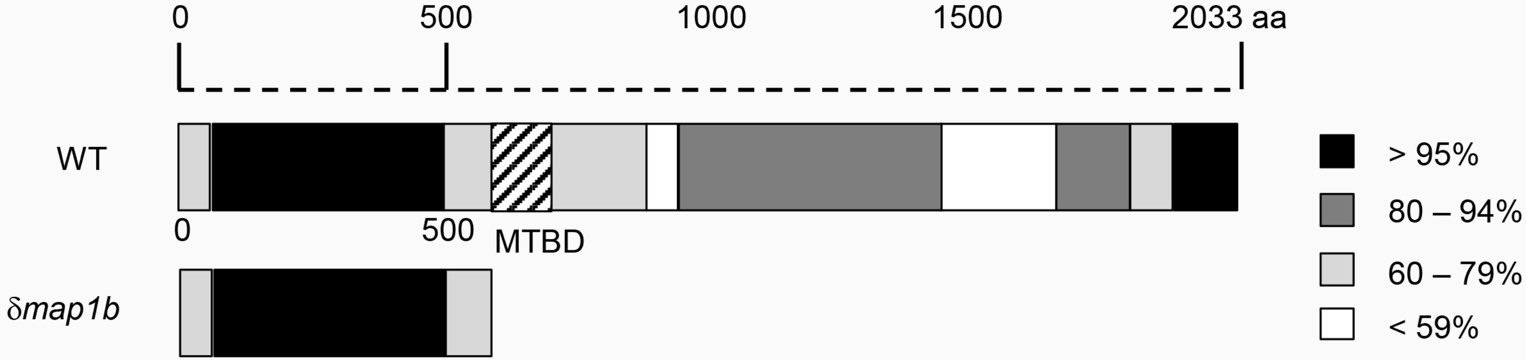


**B**

***δmap1b* RNA titration using morphology of 24 hpf as readout**

| δmap1b conc | WT | Mild | Moderate | Severe | Very severe |
|---|---|---|---|---|---|
| Uninjected | 100% | 0% | 0% | 0% | 0% |
| 25 ng/ml | 65% | 20% | 10% | 5% | 0% |
| 50 ng/ml | 48.5% | 9.1% | 12.1% | 18.2% | 12.1% |
| 75 ng/ml | 7.7% | 15.4% | 0% | 38.5% | 38.5% |
| 100 ng/ml | 0% | 0% | 27.3% | 54.5% | 18.2% |

**C**

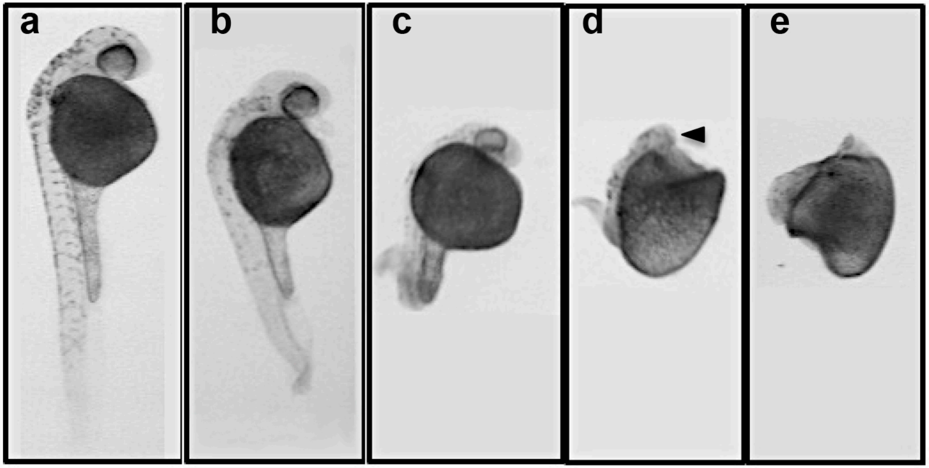

Supplement: Additional file 6: Figure S4. — δmap1b construct and RNA titration. Representation of zebrafish full length Map1b and δMap1b protein indicating percent amino acid similarity to human ortholog. Black represents the highest level of homology and white, the lowest. Hatch marks indicate the MT-binding domain (MTBD). (B) Titration analysis of δmap1b RNA. Percent of embryos with WT, mild, moderate, severe and very severe phenotypes are indicated in the table for each concentration of RNA. (C) 24 hpf embryos correspond to the phenotypic categories in the table (a: WT, b: mild, c: moderate, d: severe e: very severe). Black arrowhead indicates missing eye. (PDF 2953 kb) [file 13064_2015_56_MOESM6_ESM.pdf]
